# Counting dense object of multiple types based on feature enhancement

**DOI:** 10.3389/fnbot.2024.1383943

**Published:** 2024-05-16

**Authors:** Qiyan Fu, Weidong Min, Weixiang Sheng, Chunjiang Peng

**Affiliations:** ^1^School of Mathematics and Computer Science, Nanchang University, Nanchang, China; ^2^Institute of Metaverse, Nanchang University, Nanchang, China; ^3^Jiangxi Key Laboratory of Smart City, Nanchang, China; ^4^Jiangxi Justice Police Vocational College, Nanchang, China

**Keywords:** dense object of multiple types, crowd counting, vehicle counting, density map regression, feature enhancement

## Abstract

**Introduction:**

Accurately counting the number of dense objects in an image, such as pedestrians or vehicles, is a challenging and practical task. The existing density map regression methods based on CNN are mainly used to count a class of dense objects in a single scene. However, in complex traffic scenes, objects such as vehicles and pedestrians usually exist at the same time, and multiple classes of dense objects need to be counted simultaneously.

**Methods:**

To solve the above issues, we propose a new multiple types of dense object counting method based on feature enhancement, which can enhance the features of dense counting objects in complex traffic scenes to realize the classification and regression counting of dense vehicles and people. The counting model consists of the regression subnet and the classification subnet. The regression subnet is primarily used to generate two-channel predicted density maps, mainly including the initial feature layer and the feature enhancement layer, in which the feature enhancement layer can enhance the classification features and regression counting features of dense objects in complex traffic scenes. The classification subnet mainly supervises classifying dense vehicles and people into two feature channels to assist the regression counting task of the regression subnets.

**Results:**

Our method is compared on VisDrone+ datasets, ApolloScape+ datasets, and UAVDT+ datasets. The experimental results show that the method counts two kinds of dense objects simultaneously and outputs a high-quality two-channel predicted density map. The counting performance is better than the state-of-the-art counting network in dense people and vehicle counting.

**Discussion:**

In future work, we will further improve the feature extraction ability of the model in complex traffic scenes to classify and count a variety of dense objects such as cars, pedestrians, and non-motor vehicles.

## 1 Introduction

Dense object counting is mainly used to calculate the number of dense objects in images or videos, such as the number of vehicles in traffic jam scenes (Min et al., 2022), the crowd counting in congested public scenes (Zhao et al., 2021), the number of specific bacteria or cells in microscopic scenes (Fan et al., 2022), and the goods on shelves in indoor packed scenes (Goldman et al., 2019). The achievements of dense object counting research have been widely used in the fields of traffic flow prediction, public safety management, biology and medicine-pharmacy, and supermarket monitoring and management. The research is also an integrated multidisciplinary research direction, including artificial intelligence, computer vision, machine learning, deep learning, and pattern recognition (Dirir et al., 2021). Therefore, dense object counting has become one of the research hotspots with significant attention and development potential in recent years.

In 2010, Lempitsky and Zisserman (2010) first proposed using the density map regression method to count objects in an image. This method avoided the task of learning to detect and segment individual count object instances, effectively solved the problem of mutual occlusion of dense objects, and thoroughly considered the spatial features of the image. The core idea is to convert the counting problem to estimate the density of an input image and then integrate the predicted density map to get the number of objects in that image region. In recent years, with the advent of deep learning, Convolution Neural Networks (CNN) have achieved great success in various computer vision tasks, so for dense object counting tasks, researchers are also gradually using CNN to extract features and complete the prediction from the input image to the density map. These methods (Fu et al., 2015; Walach and Wolf, 2016; Zhang et al., 2016; Sam et al., 2017; Li et al., 2018) based on CNN can handle severe occlusions in dense object images, generate accurate density maps, and perform better than traditional methods based on manual features. Dense object counting methods based on density map regression are divided into two categories: the traditional methods based on manual features (Rodriguez et al., 2011; Fiaschi et al., 2012; Arteta et al., 2014; Ma et al., 2015) and the CNN-based methods (Fu et al., 2015; Walach and Wolf, 2016; Zhang et al., 2016; Sam et al., 2017; Li et al., 2018). In traditional methods based on manual design features, the commonly used features are image gray value, color value, and SIFT features (Rodriguez et al., 2011; Ma et al., 2015); commonly used regression models include ridge regression (Arteta et al., 2014) and random forest regression (Fiaschi et al., 2012). Compared with traditional methods, the density map object counting method based on CNN does not need to select image features manually and enables the model to learn end-to-end through training, which has better counting performance. The reason is not only because of the density map regression framework’s advantages but also because deep learning models need much supervised information to learn. The density map regression method can provide more supervisory information.

In the dense object counting task, the CNN-based method can obtain excellent counting performance, and the research based on the CNN network has also produced many excellent algorithm models (Fu et al., 2015; Walach and Wolf, 2016; Zhang et al., 2016; Sam et al., 2017; Li et al., 2018), which can obtain satisfactory counting results in many application scenarios. However, the task still needs targeted research on the following problems. First, most of the current dense object counting algorithms are only applicable to a single class of object counting in simple scenes, such as dense vehicles in traffic jam scenes or packed crowds in public scenes, while the objects in natural scenes are complex, such as crowds and vehicles are mixed. Therefore, the counting model needs more research on multiple types of dense objects, such as the simultaneous counting of crowds and vehicles in complex traffic scenes. Secondly, the background of the people-vehicles mixed traffic environment is often complex, so enhancing the spatial and channel features of the counting objects is necessary. The spatial features are mainly embodied in the spatial correlation features, that is, the perspective relationship and scale change between the counting objects, while the channel features are mainly manifested in the channel salience features, that is, the types of the counting objects. In addition, the current counting model usually uses Euclidean distance as a loss function to measure the difference between the predicted density map and the ground truth (GT) density map. Euclidean distance loss assumes the independence of pixels while ignoring the local spatial correlation between the GT density maps. The Euclidean distance loss function is also more sensitive to abnormal sample points and fuzzy images.

To alleviate these problems, we propose a novel multiple types of dense object counting method to achieve the classification counting of dense vehicles and crowds in complex traffic scenarios and train the model with a new joint loss function. The multiple types of dense object counting method adopts CNN as the primary network model. It uses the auxiliary loss function to help realize the task of multiple types of dense object classification and density map regression. The network model is an end-to-end cascading framework that generates high-quality 3D predicted density maps that calculate dense vehicles and people in the image. First, the image to be predicted is sent to the convolution layers of VGG for the extraction of initial image features (Chen et al., 2020), then the feature enhancement layer (FEL) is used to enhance the initial features and extract deeper spatial correlation and channel significance information, and finally, deconvolution layers are used to complete the upsampling and generate the predicted two-channel 3D density map. This predicted density map shows classification and distribution information, and it contains two channels, one representing the probability distribution of counting vehicles and the other being the probability distribution of counting people; thus, multiple types of dense object counting of vehicles and people can be achieved simultaneously. In this study, the classification subnet is used to supervise the classification task, and the auxiliary loss functions *L_cla_* and *L_xy_* are designed to generate two-channel heat maps, which are divided into two categories: vehicles and people. Each channel heat map represents the probability distribution of the center point of a class of counting objects. However, the classification task does not output predicted results but completes the two-channel classification supervision of multiple types of dense object counting tasks. Then, the regression of the two-channel density map is completed through the loss function *L_cou_* of the main task, and the accurately predicted density map is generated to complete the count of dense vehicles and crowds. The experimental results based on the benchmark dataset show that the proposed method performs better than the existing methods.

The main contributions of this study are summarized as follows:

1. The proposed method enhances spatial correlation information for density map regression and channel saliency information for classification to achieve the counting of different types of dense objects (vehicles and people) in complex traffic scenarios. FEL is mainly used to enhance the spatial and channel features of the foreground counting object in a complex background. The spatial correlation module (SCM) completes enhancing spatial correlation features based on perspective change effects and dense multi-scale variation, and the channel saliency module (CSM) obtains the saliency coefficient of each channel by learning. The suppression of complex background channel features and the enhancement of foreground object channel features are completed.
2. In this study, the classification subnet is used to implement the channel classification supervision of the model to assist in the regression task of the two-channel density map and realize the simultaneous counting of dense vehicles and crowds in the same scene. The auxiliary loss functions *L_cla_* and *L_xy_* are modified Focal loss function and L1 loss function, which not only solved the supervised training of two-channel classification counting but also improved the correlation of local space ignored by using the Euclidean distance loss function (L2 loss) alone and improved the counting performance under the single channel.

The rest of this paper is organized as follows: Section 2 discusses related works. Section 3 introduces the whole method and the details of the proposed model for multiple types of dense object counting. Section 4 describes the joint loss function. Section 5 shows the experiment results, and Section 6 gives the conclusions and future research directions.

## 2 Related work

Counting tasks based on computer vision enables the computer to estimate the number of relevant objects in the image accurately. As discussed in Section 1, the approaches based on density map regression can effectively solve the occlusion problem in dense object counting. Generally, it can be divided into two categories according to different methods of feature extraction: the methods based on manual features and the CNN-based methods. In this section, related studies on dense object counting using the density map regression approaches are reviewed from two aspects: the methods based on manual features and the methods based on CNN.

### 2.1 The methods based on manual features

Lempitsky and Zisserman (2010) proposed for the first time an object counting algorithm model based on density map regression, which aims to solve the occlusion problem between objects and accurately estimate the number of interested dense objects in the image. The central processing steps of the counting method are as follows: First, the center point of the annotated counting object is processed by the Gaussian kernel function, and the ground truth image of the density distribution of the counting object is generated (also known as GT density map later). Then, the GT density maps are used as the training set label to extract image features from the input image, train the regression model, and directly learn the mapping relationship from image pixel features to GT density maps. Finally, the prediction of the density map is completed, which reflects the distribution of the object in the image. The number of targets in any region can be obtained by integrating the regional density map, and the estimated count of the objects in the image can be completed. The early counting methods all adopted manual features, and the commonly used features were image gray value, color value, and SIFT features (Rodriguez et al., 2011; Ma et al., 2015). The regression model often used the ridge regression model (Arteta et al., 2014) and the random forest regression model (Fiaschi et al., 2012). In 2016, Wang and Zou (2016) designed a density map estimation algorithm based on subspace learning, which can effectively solve the problem of low computational efficiency caused by traditional manual feature extraction. These methods use traditional manual features to complete the extraction of low-level spatial information, which is inefficient and cannot guide the generation of high-quality predicted density maps to obtain more accurate dense object counting.

### 2.2 The methods based on CNN

Recently, deep learning has achieved great success in various computer vision tasks, especially CNN, which is widely used for its robust feature representation capabilities. In 2015, Fu et al. (2015) introduced deep learning into the object counting task based on density map regression, using the CNN-based model to count dense crowds. Compared with the traditional manual feature methods, this method has significantly improved the counting performance. The CNN-boosting model (Walach and Wolf, 2016) adopts CNN as the primary network in a layer-wise manner, making full use of layered boosting and selective sampling to improve the performance of the counting model and improve training efficiency. Inspired by the research on multi-column deep neural networks in multi-scale problems, Zhang et al. proposed a density map regression model MCNN (Zhang et al., 2016) with multi-column networks. The MCNN uses different kernel sizes (large, medium, and small) in each network column to capture the features of objects at different scales in the image. The Switching CNN model (Sam et al., 2017) trains several independent CNN density regressors that have the same structure as the MCNN (Zhang et al., 2016) and uses a switching classifier to select the best one for density map estimation. These models usually use different columns to capture multi-scale information corresponding to different receptive fields, which makes the dense object counting have good performance, but at the same time, increases the complexity and training difficulty of the network and produces much redundant information. CSRNet (Li et al., 2018) uses a dilated convolutional layer to extend the receptive fields while maintaining resolution as the back-end network. Instead of using a multi-column architecture, the basic idea is to design a deeper CNN to obtain higher-level features with larger receptive fields and generate high-quality density maps without expanding the complexity of the network. These single-column networks usually have a deeper network structure, reduce the complexity of the network, and have good counting performance. Zhou et al. (2020) proposed a shallower single-column CSCNet, whose fundamental functional structure can capture complementary scale context information and obtain high counting accuracy without deepening the depth of the network. In addition to studying the improvement of the network structures, the design of appropriate loss functions is also an important factor that directly affects the performance of counting models. The density map regression methods often use Euclidean distance as a loss function for training regression models. However, some problems can still be continuously improved, such as sensitivity to outliers (represented as isolated counting objects). Furthermore, the Euclidean distance loss ignores the local spatial correlation between the predicted density map and the GT density map. Furthermore, the assumption of pixel independence using Euclidean distance loss ignores the density map’s local coherence and spatial correlation. Therefore, Jiang et al. (2019) designed a joint loss function, including the spatial abstract loss (SAL) and the spatial correlation loss (SCL), which takes into account the spatial correlation between pixels to improve the prediction quality of density maps. Dense object counting requires manual point annotation of training labels. Gao et al. (2023) proposed a domain adaptive crowd counting framework to solve the problem of crowd counting labels relying on many manual labels and further refining the quality of density maps on accurate data. Wang et al. (2023) proposed a new dynamic counting network hybrid approach from the perspective of different learning paradigms in order to solve the problem that counting labels requires a large number of manual point annotations. In addition, Gao et al. (2021) proposed a domain-adaptive style counting method, which mainly addresses the problem of model performance degradation caused by the domain gap between realistic images and synthetic images. To address the gap between complex network architectures pursuing high-precision counting and limited computing and storage resources, Yi et al. (2024) propose a lightweight crowd counting network based on an encoder-decoder to achieve an optimal trade-off between counting performance and running speed. However, these methods count the crowd or dense vehicles separately in a single scene and do not consider the problem of counting multi-class objects in complex traffic scenes. Some models study the counting of multi-class objects. Gao et al. (2024) proposed to study the counting of multi-class objects (cars, buildings, ships, etc.) in an aerial image. This counting model uses a dual attention module to integrate the features of RGB and NIR. A large-scale dataset (NWPU-MOC) is built for this task. This method counts multi-class objects from the perspective of aerial photography and does not study the counting of complex foreground objects in complex traffic backgrounds. Xu et al. (2021) simultaneously estimated vehicles and pedestrians in a single image, designed DSAM for capturing multi-scale information, and CAM for adaptive suppression of inter-class interference during feature extraction to complete classification and counting, but did not consider the perspective effects of dense objects. Our method mainly realizes the simultaneous counting of two types of objects (vehicles and crowds) in complex traffic scenes, enhancing objects’ spatial features that consider perspective effects and multi-scale variations and channel features for auxiliary classification in complex traffic environments. Feature enhancement is used in computer vision tasks such as object detection and Semantic Segmentation. Zhang et al. (2023) propose a spatial attention model to highlight the saliency of the target of interest and use two sets of feature maps of different scales to construct context connections and complete feature enhancement in the spatial domain for ship detection. Liu et al. (2023) proposed a feature enhancement module to suppress interference caused by local similarity between classes and enhance semantically relevant response features on the channel domain. Our method carries out feature enhancement in both dimensions. In the spatial domain, it solves the continuous large-scale scale variation and perspective effects of counting objects. In the channel domain, the salience coefficient of each channel is obtained by learning, which is mainly used to suppress complex background information and enhance the classification features of the foreground targets to achieve the counting of the two types of objects. In addition, we also fully consider the spatial correlation lost by using Euclidean distance loss in density map regression. In this paper, the classification subnet is designed to help supervise two-channel (vehicle and people) classification, and the joint loss function is used to enhance the spatial correlation and improve the quality of the predicted density map.

## 3 Our method

In this study, a multiple types of dense object counting network is used to predict the density map of two types of counting objects (crowd and dense vehicles), and the number of people and vehicles in the image is counted by integrating the density map. In this section, we summarize the method of multiple types of dense object counting, describe the structure and function of the counting network in detail, and introduce the two-channel GT density map.

### 3.1 Overview of the method

**Figure 1:**
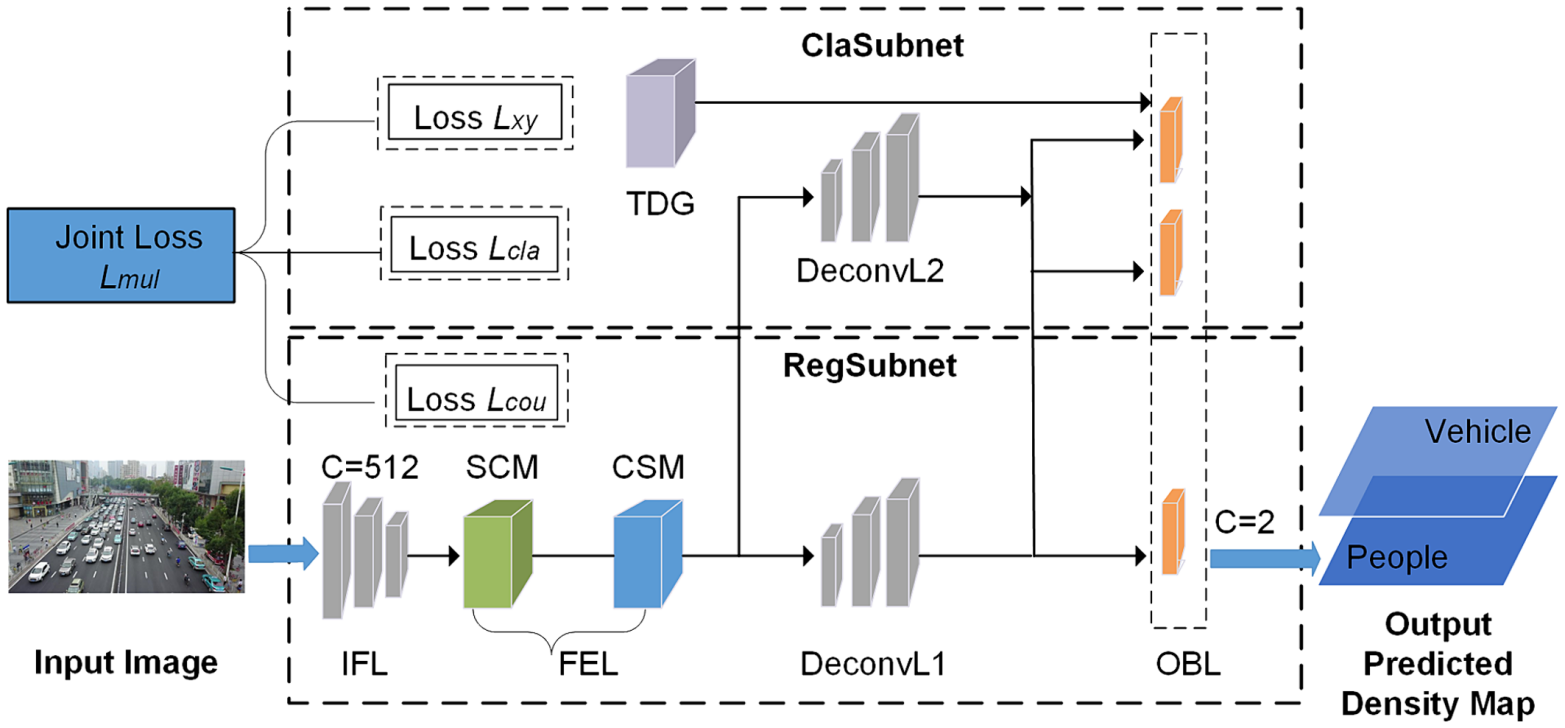
The architecture of the proposed method.

The counting network is a deep CNN network using a coding-decoding (Li et al., 2018) structure, which can generate high-quality two-channel predicted density maps to complete the count of people and vehicles in complex environments. The input image is processed by the initial feature layer (IFL), the feature enhancement layer (FEL), and the deconvolution layer (DeconvL), and then a two-channel predicted density map is output through the output branch layer (OBL). The counting network includes a classification subnet (ClaSubnet) and a regression subnet (RegSubnet). Among them, the ClaSubnet mainly completes the auxiliary supervision task of classifying vehicles and people by channel, and the regression subnet RegSubnet completes the main density map regression task, predicting the high-quality two-channel density map (vehicle and people). Finally, the estimated number of people and vehicles in the image can be obtained by integrating the two-channel predicted density map. The counting method architecture is shown in Figure 1.

The IFL has a strong transfer learning ability and is used to extract the initial features of the image. The FEL is used to extract deeper information about saliency and enhance the initial features. FEL consists of two functional modules, the spatial correlation module (SCM) and the channel saliency module (CSM), which are mainly used to enhance the features of vehicles and people in complex traffic environments. The SCM module completes enhancing spatial features based on perspective effects and dense multi-scale variation. By learning the importance of different feature channels, the CSM module obtains the significance coefficient of each channel, which is used to enhance the counting features of foreground objects in complex backgrounds, and these features contain the channel saliency information needed to assist the classification task. The DeconvL upsamples the feature map. The two-channel GT Density Map generator (TDG) is mainly used to generate two-channel ground truth density maps as training labels for counting tasks. The OBL consists of three branches. The main task branch generates the two-channel predicted density map, and the auxiliary task branches help classify the object and confirm the offset of the object’s center point. This method uses joint loss functions *L_mul_*, including *L_xy_*, *L_cla_*, and *L_cou_*, where *L_cou_* is used for density map regression training for the main task, and *L_xy_* and *L_cla_* are used for classification training for the auxiliary task. The whole method is an end-to-end training process. The input image can be converted to the high-quality two-channel predicted density map directly and get the number of people and vehicles.

### 3.2 RegSubnet

**Figure 2:**
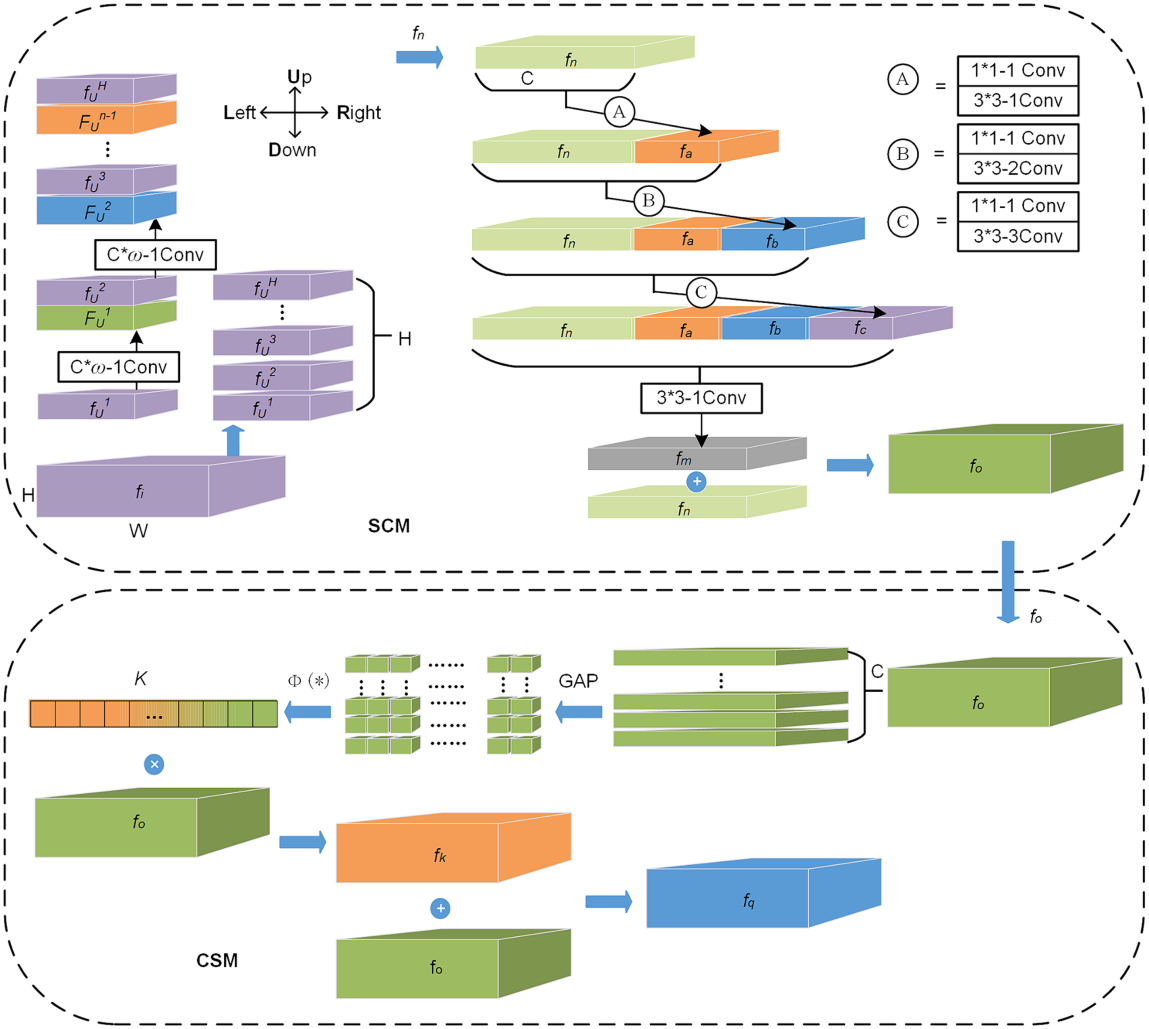
The structure of the FEL.

The RegSubnet completes the main counting task, the regression task of the density map, using the Loss *L_cou_* to train the network to output the predicted density map of the two channels (vehicle and people). The main functional layers of the subnet are as follows:

- The IFL uses the first 13 layers of the VGG-16 (Simonyan and Zisserman, 2014) network, which has powerful analytical capability for dense objects and is used as the backbone network by most dense object counting models. We use transfer learning to improve the training efficiency of our model. However, IFL extracts only the initial features of the image because it encodes only the limited receptive field of the input image. We deployed FEL to enhance the features of the counting target, encode a broader range of receptive fields, and extract deeper spatial correlation information and channel saliency information.
- The FEL consists of two functional modules, the spatial correlation module (SCM) and the channel saliency module (CSM), mainly used to enhance the features of vehicles and people in complex traffic environments, and its structure is shown in Figure 2. Through the research of related work in Section 2, the main problems to be solved in the complex traffic environment are perspective effects, scale variation, complex background environment, and complex foreground object. We use the FEL layer to solve these problems effectively, in which the SCM module extracts features from four directions. The perspective information hidden between the rows and columns of the feature map is extracted. Then, the superimposed atrous convolution groups are used to obtain a wide range of receptive fields for multi-scale feature fusion. The module mainly completes enhancing spatial features based on perspective effects and dense multi-scale variation. The CSM module can learn the importance of different feature channels, obtain the saliency coefficient of each channel to weigh all channel features and enhance the counting features of the foreground object in the complex background. These features contain the channel saliency information needed to assist the classification task. This module mainly suppresses complex background channel features and enhances the channel features of the foreground object.
- The DeconvL1 consists of three deconvolution groups, each including a 3 × 3 convolution and a deconvolution. Each deconvolution operation will double the size of the feature map and complete the upsampling of the feature map.
- The OBL consists of three branch convolutional networks. The main task branch is used to generate the two-channel predicted density map, and the other two auxiliary task branches belong to the classification subnet, which are used to supervise the two-channel classification of people and vehicles and the offset of the counting object center point.

#### 3.2.1 SCM

SCM module is mainly used to enhance spatial correlation information in complex traffic environments. The module finely encodes the feature map after initial feature extraction in four directions (U, D, L, and R) on the length and width planes. This operation extracts as much spatial information as possible that is hidden due to perspective distortion effects, and in the counting task, the density distribution features of vehicles or people are also included in this spatial information (Zhao et al., 2021). As shown in Figure 2, the SCM module first slices the input feature map *f_i_* from bottom to top in the H direction. Then, the first feature slice, *f_U_^1^*, performs the convolution layer operation. Each convolution layer mainly consists of a convolution operation of ω × C size and a ReLU activation function. The module then adds (element-by-element) the features *F_U_^1^* processed by the convolutional layer to the next feature slice *f_h_^2^* to form a new output and continues to carry out convolutional layer operations until all feature slices have completed convolution and superposition operations, which we name the U direction operation, as shown in Equation (1):


(1) $$F_{U}^{i}=F_{U}\left(F_{U}^{i-1}+f_{U}^{i}\right),i=1,2,3\ldots \ldots .H$$

where *F_U_* (*) represents the U direction operation, *f_U_^i^* is the ith feature slice in the U direction. Similarly, the operation in the other three directions (D, L, and R) performs the same except for the direction of the feature slice and the sliding direction of the convolution kernel. After completing the operation of the four direction convolution layers successively, the enhanced feature *f_n_* is obtained. Then, the SCM module inputs *f_n_* into three atrous convolution layers (Ⓐ, Ⓑ, and Ⓒ) with different dilation rates and obtains seven sizes of receptive fields by concatenating features on channel dimensions. This operation can complete the enhancement of multi-scale features in space. As shown in Figure 2, *f_n_* first obtains feature fa through the operation of layer Ⓐ, which mainly includes a 3 × 3 convolution kernel with a dilation rate of 1. Then *f_a_* and *f_n_* are concatenated in channel dimension and input to layer Ⓑ for atrous convolution operation. The dilation rates of the atrous convolution of layers Ⓐ, Ⓑ, and Ⓒ are set to 1, 2, and 3, respectively. A standard 1 × 1 convolution is added before each atrous convolution, controlling the number of channels, and the ReLU activation function is used after each atrous convolution. In this module, the method to efficiently use spatial multi-scale information for feature enhancement is to add all captured multi-scale fusion feature *f_m_* and original feature *f_n_* element-by-element, as shown in Equation (2).


$$f_{o}=f_{n}+f_{m}$$

(2) $$f_{m}=\mathrm{Con}\left(\left[f_{n},f_{a},f_{b},f_{c}\right]\right)$$

The function *Con*(*) represents the connection of the four features *f_n_*, *f_a_*, *f_b_*, and *f_c_* in the channel dimension and uses the standard convolution of 3 × 3 to complete the channel fusion. The operation shown in Equation (2) combines and stacks the atrous convolution and transfers and obtains the receptive fields of various sizes through channel concatenating to enhance the spatial relationship of the feature maps of different scales and retain the initial spatial feature information. The SCM module can effectively encode the perspective information of dense objects from four directions and obtain the spatial relationship information of multi-scale feature maps. It is especially suitable for extracting the features of dense vehicles and people with strong spatial correlation, but the actual appearance clues are not obvious.

#### 3.2.2 CSM

SCM enhances features in the spatial dimension, while CSM enhances features in the channel dimension. CSM module is mainly used to enhance channel saliency information in complex traffic scenes. This module can learn saliency information about important channels. Specifically, the model obtains the saliency coefficient of each channel through learning and then uses these coefficients to enhance the classification features of the counting target and suppress the unimportant channel features for the classification task, as shown in Equation (3):


$$f_{q}=f_{o}+K⊙f_{o}$$

(3) $$K=\omega \left(\mathrm{GAP}\left(f_{o},C\right)\right)$$

where *K* represents the saliency coefficient, the function ω (*) consists of a bottleneck network with two fully connected layers, as well as the ReLU and Sigmoid functions. As shown in Figure 2, the enhanced feature *f_o_* after SCM processing is input into CSM, which is first stratified according to channel. Then, global average pooling (GAP) is performed for each layer to obtain global information and introduce attention to channel dimensions. After C 1 × 1 feature maps are obtained, the function ω (*) is used to process these feature maps to get channel significance coefficient *K*. By introducing GAP, the module ignores the correlation in spatial distribution and pays more attention to the significance information of channels. Through the activation function Sigmoid in ω (*), the significance coefficient *K* of each channel is obtained. The coefficient *K* of each channel is then multiplied with the original input feature *f_o_* to enhance the features on the channel dimension. Finally, the enhanced feature *f_x_* is added to the input feature *f_o_* to enhance further the feature maps’ space and channel representation capability (Szegedy et al., 2017). The significance coefficient *K* reflects the activity of channels, and some channels are in a highly activated mode, which is related to the features of different kinds of counting objects that need to be learned. Therefore, the CSM can enhance the features of classification tasks, especially the salient features of vehicles and crowds in complex traffic scenes.

The network’s final output is an estimated density map of two channels, one of which predicts the number of people and the other predicts the number of vehicles.

### 3.3 ClaSubnet

The ClaSubnet mainly completes the auxiliary supervision function of classification tasks and use auxiliary loss functions *L_cla_* and *L_xy_* to classify dense vehicles and people by channel. Its functional structure is shown in Figure 1, which mainly includes the DeconvL2, the OBL, and the two-channel GT density map generator (TDG).

**Figure 3:**
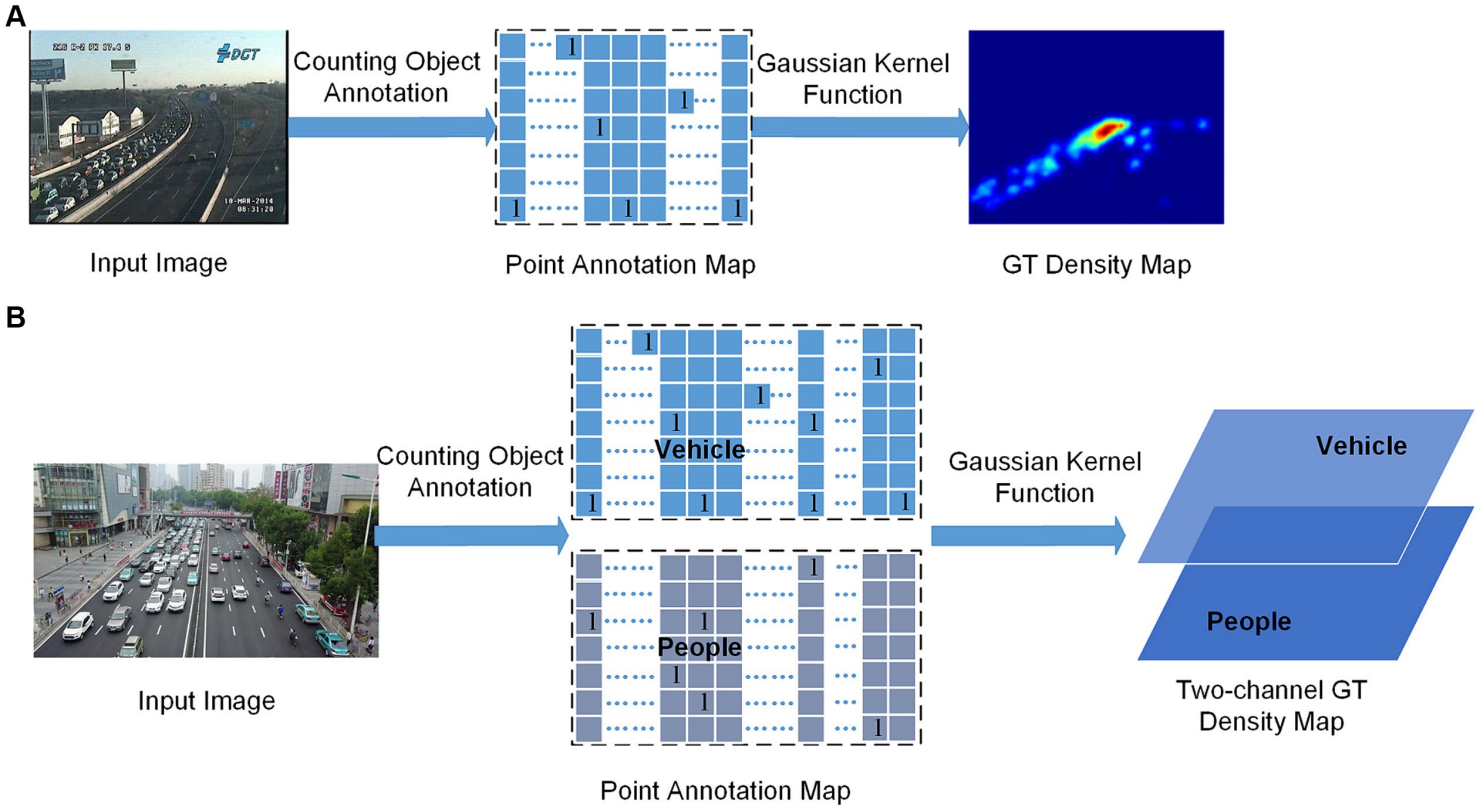
Generation of one-channel **(A)** and two-channel **(B)** GT density maps.

- The DeconvL2 has the same network structure as the deconvolution in the RegSubnet, mainly including 3 × 3 convolutions and deconvolutions to complete the upsampling of the image.
- The OBL consists of three branch convolutional networks. The two auxiliary task branches belong to the ClaSubnet. These two auxiliary task branches can obtain the predicted heat map and offset of the object’s center point but do not output the prediction. They are primarily auxiliary supervisors to help complete the classification count of dense vehicles and crowds and improve the spatial correlation of predictions.
- The TDG is mainly used to generate two-channel GT density maps as training labels for learning tasks. The GT density map mainly uses the Gaussian kernel function (Lempitsky and Zisserman, 2010) to generate the probability density value corresponding to the point annotation and surrounding pixels of the counting object, and the probability sum of one counting object area is 1. We can obtain a two-dimensional density map of the vehicle or crowd as the training label. In this paper, the GT generation process of the density map label is optimized, and a two-channel three-dimensional GT density map is generated as the training label, hoping to represent better the density map of different counting objects in the mixed scene, as shown in Figure 3.

In Figure 3, A represents the generation of the traditional one-channel GT density map, which is used as a training label to estimate a class of objects (people or vehicles). B represents the generation of the two-channel GT density map used in this paper, which can be used as a training label to estimate two types of objects (people and vehicles). Whether A or B, the center of the counting object needs to be annotated first, like the PA (point annotation) map in the figure, but the PA map in the figure contains only one pixel for each object (typically the center of the people’s head and vehicle), which is sparse, and contains no information about the object size and shape. It is not feasible to train the predicted density map directly using this sparse point annotation map. A common remedy for this difficulty is converting it to a GT density map using the Gaussian kernel function. When generating the traditional GT density map, it is necessary first to generate the PA map. In this study, the PA map is an intermediate step in generating GT density maps and is also used to generate training labels for auxiliary tasks. This is shown by B in Figure 3, a two-channel PA map will be generated first, and then a two-channel GT density map will be generated by a Gaussian kernel function.

Let I ∈ R*^W × H × 3^* be an input image of width W and height H. Our aim is to produce a two-channel GT density map G(x_m_) ∈[0, 1) ^W/r × H/r × 2^, Where *r* is set to 2 according to the size of the output predicted density map and 2 is the number of counting object types. Let G(x_m_) = {x_m_ | 0 ≤ D_GT_(x_m_) < 1: m = 1, 2,..., *M*} be a density map, where x_m_ denotes a 2D-pixel location, and *M* is the number of pixels in the density map. Let P(x_m_) = {x_m_ | (x_m_ ≠ x_n_, 0), (x_m_ = x_n_, 1): n = 1, 2,..., *N*} denote the counting point annotation map for an input image, where *N* is the total object count, x_n_ is a 2D counting point position, and x_m_ still represents the position of a 2D pixel. D_GT_(x_m_) is represented by the following Equation (4):


(4) $$\begin{array}{c} D_{GT}\left(x_{m}\right)\overset{\;def\;}{=}\overset{N}{\underset{n=1}{\sum }}N\left(x_{m};x_{n},\sigma^{2}1_{2\times 2}\right)\\ =\overset{N}{\underset{n=1}{\sum }}\frac{1}{\sqrt{2\pi }\sigma }\exp\left(-\frac{||x_{m}-x_{n}||{\;}_{2}^{2}}{2\sigma^{2}}\right) \end{array}$$

where N (x_m_; x_n_, σ^2^1_2 × 2_) denotes a 2D Gaussian distribution evaluated at pixel location x_m_, with the mean at the annotated point x_n_, and where σ is an object size-adaptive standard deviation, and the value is determined by the counting object. In this work, the vehicle object is set to 15, and the crowd object is set to 10.

This study does not directly use the PA map to supervise the auxiliary task. However, it uses a mixed Gaussian distribution of multiple annotated points in the PA map as training labels. The predicted heat map center points can be located in these Gaussian distribution regions. The Gaussian distribution is expressed in Equation (5):


(5) $$\begin{array}{c} D_{PA}\left(x_{m}\right)\overset{\;def\;}{=}\overset{N}{\underset{n=1}{\sum }}N\left(x_{m};,x_{n};,\mu^{2}1_{2\times 2}\right)\\ =\overset{N}{\underset{n=1}{\sum }}\exp\left(-\frac{||x_{m}-x_{n}||{\;}_{2}^{2}}{2\mu^{2}}\right) \end{array}$$

where N (x_m_; x_n_, μ ^2^1_2 × 2_) denotes a 2D Gaussian distribution evaluated at pixel location x_m_, with the maximum 1 at the annotated point x_n_, and where μ is a standard deviation related to the size of the object, set to 5 for people and 7.5 for vehicle in this study.

## 4 Joint loss function

The predicted output of our method is the two-channel estimate density map. This estimated density map contains two channels, one for vehicle density prediction and the other for people density prediction. The number of vehicles and crowd is obtained by integrating the estimated density maps of the two channels. In this study, a joint Loss *L_mul_* based on auxiliary tasks is used to complete the task of channel classification and counting. *L_cou_* is used for the density map regression task of the RegSubnet, and *L_cla_* and *L_xy_* in the ClaSubnet mainly completed the task of classifying by channel. The joint Loss *L_mul_* is shown in Equation (6):


(6) $$L_{mul}=L_{cou}+\lambda_{I}L_{cla}+\lambda_{2}L_{xy}$$

where *L_cou_* is the L2 loss function, which is used for the training of the main task density map regression. The *L_cla_* is set up with reference to the Focal loss (Lin et al., 2020) function to supervise the two-channel classification of auxiliary tasks. According to the comparison of experimental results, the value of *λ_1_* = 0.8 is the best. *L_xy_* is the L1 loss function, which is used to supervise the position offset of the center point of the counting object in the two-channel classification of auxiliary tasks, and the same class of counting objects share the same offset prediction.

*L_cou_* loss function as shown in Equation (7):


(7) $$L_{cou}=\overset{M}{\underset{m=1}{\sum }}F\left(D_{GT}\left(x_{m}\right)-{\hat{D}}_{GT}\left(x_{m}\right)\right)$$

where *F*(·) is a distance function and ${\hat{D}}_{GT}\left(x_{m}\right)$ is the predicted density map.

*L_cla_* loss function as shown in Equation (8):


(8) $$L_{cla}=-\frac{1}{N}\sum x_{m}\{\begin{array}{c} \begin{array}{l} \beta {\left(1-{\hat{D}}_{PA}\left(x_{m}\right)\right)}^{\alpha }\cdot \log\left({\hat{D}}_{PA}\left(x_{m}\right)\right)\;\\ \mathrm{if}\;D_{PA}\left(x_{m}\right)=1 \end{array}\\ \begin{array}{l} \left(1-\beta \right){\left({\hat{D}}_{PA}\left(x_{m}\right)\right)}^{\alpha }\cdot \log\left(1-{\hat{D}}_{PA}\left(x_{m}\right)\right)\;\\ \mathrm{otherwise}\; \end{array} \end{array}$$

where *D_PA_*(x_m_) and ${\hat{D}}_{PA}\left(x_{m}\right)$ are the Gaussian distribution of the PA map and the predicted heat map; α and β are hyper-parameters of the *L_cla_*, and *N* is the number of counting points in the image.

*L_xy_* loss function as shown in Equation (9):


(9) $$L_{xy}=\frac{1}{N}\overset{N}{\underset{k=1}{\sum }}|{\hat{O}}_{\;\tilde{c\;}}-\left(\frac{C}{2}-\tilde{C\;}\right)|$$

where C represents the central point coordinates of the counting object, C/2 indicates that the downsampling multiple after codec is 2, and $\tilde{C}$ is C/2 after rounding, representing the loss value of the coordinates of the central point. ${\hat{O\;}}_{\tilde{C}}$ is the predicted result of the subnetwork.

A prediction ${\hat{D}}_{PA}\left(x_{m}\right)=1$ corresponds to a detected counting point. The normalization by *N* is chosen to normalize all positive focal loss instances to 1. When *D_PA_*(x_m_) is used as a classification training label, the number of negative samples easily classified is too large due to the imbalance between positive and negative samples, which will make the optimization direction of the model different from what we hope. *L_cla_* loss function reduces the weight of easily classified samples and makes hard training samples play a leading role; that is, the change of model parameters is mainly in the direction of optimizing hard-classified samples. In *L_cla_*, α is used to reduce the loss contribution of well-classified samples and focus the training center of gravity on hard-classified samples (Lin et al., 2020); *β* is used to adjust the ratio of loss between the positive and negative samples to solve the problem of positive and negative samples imbalance. We use *α* = 2 and *β* = 0.2 in all our experiments.

## 5 Experiments

### 5.1 Datasets and evaluation metrics

This work compared the state-of-the-art methods on three public datasets, including VisDrone (Zhu et al., 2019), ApolloScape (Gao et al., 2020), and UAVDT (Liu et al., 2018), to evaluate the effectiveness and superiority of our method. Table 1 shows the statistics of these datasets.

**Table 1:** Statistics of different datasets.

Datasets	Images	Count statistics			Average resolution
		Max	Min	Total	
VisDrone+	2,949	425	10	198,843	991 × 1,511
ApolloScape+	4,914	469	10	308,987	2,710 × 3,384
UAVDT+	9,334	590	10	399,986	540 × 1,080

**Figure 4:**
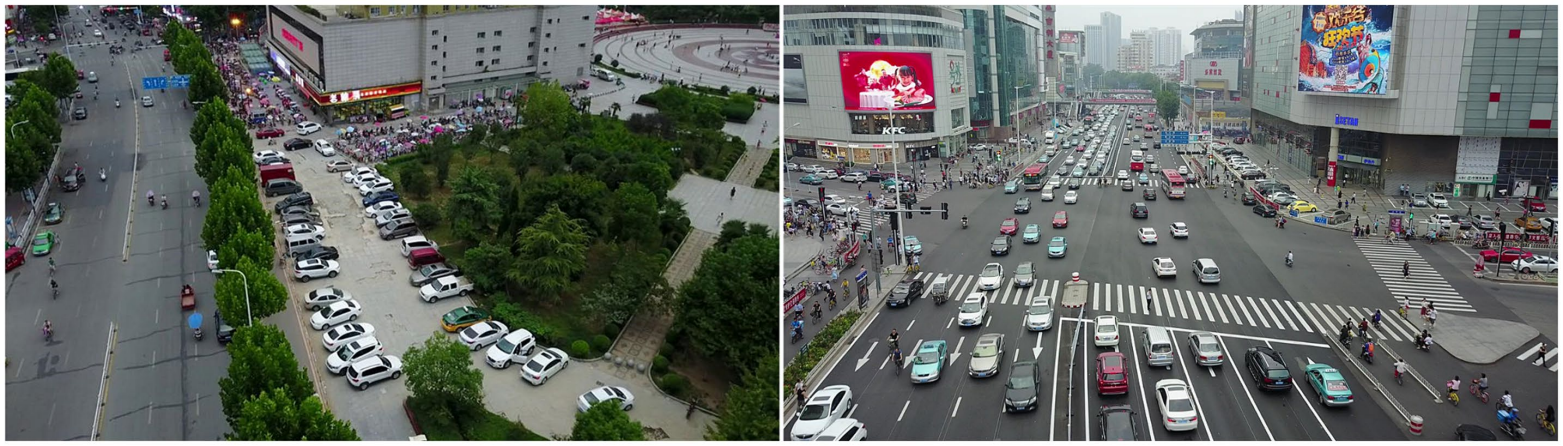
Images from the VisDrone dataset.

The VisDrone dataset is derived from an object detection dataset with annotated bounding boxes for the targets. We modified the original VisDrone dataset to form 3D GT density map labels and conducted extensive experiments on the new dataset VisDrone+. The VisDrone dataset is an object detection dataset with an annotated bounding box, which takes the two coordinate positions of the bounding box as the location of objects. The dataset is a mixed dataset of vehicles and people, with serious occlusion between objects and different perspective changes, as shown in Figure 4. The original dataset contains 11 categories. We select category pedestrians and category people to form the people channel of the label and combine category car, category van, category truck, and category bus to construct the vehicle channel of the label. The new annotation location of the VisDrone+ dataset is the people’s head point or the vehicle’s center point. In the original dataset, the images with fewer annotated objects than ten are filtered out. Finally, the VisDrone+ dataset consists of 2,019 training images, 302 validation images, and 628 test images. The new crowd channel annotation operation is defined as Equation (10).


(10) $$\mathrm{People}=\left[bbox_{\mathrm{left}}+\;\frac{{\mathrm{bbox}}_{\mathrm{width}}}{2},{\mathrm{bbox}}_{\mathrm{top}}\right]$$

The new vehicle channel annotation operation is defined as Equation (11).


(11) $$\mathrm{Vehicle}=\left[bbox_{\mathrm{left}}+\;\frac{{\mathrm{bbox}}_{\mathrm{width}}}{2},{\mathrm{bbox}}_{\mathrm{top}}+\;\frac{{\mathrm{bbox}}_{\mathrm{height}}}{2}\right]$$

**Figure 5:**
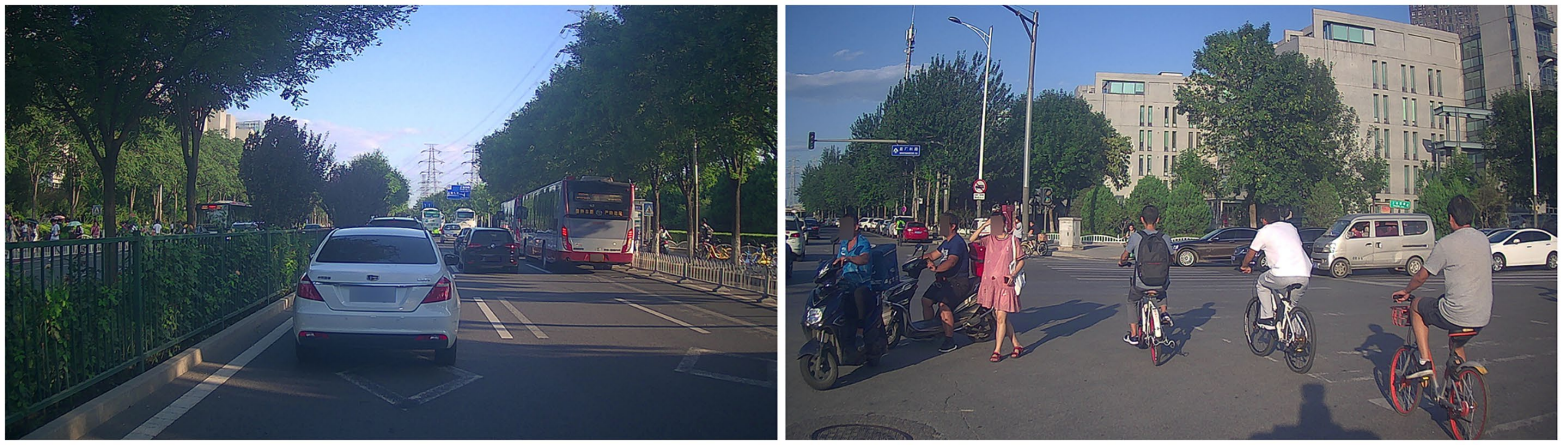
Images from the ApolloScape dataset.

The ApolloScape dataset is a large-scale street-level image dataset. It contains 147,000 annotated images with a total of 23 object categories. Annotation is performed in a dense and fine-grained style using polygons to delineate individual objects. This dataset has a complex traffic scenario, and the scale of counting objects varies greatly, as shown in Figure 5. We select category persons to form the crowd channel of the two-channel GT density map label and combine category car, category truck, and category bus to construct the vehicle channel of the label. In the original dataset, the images with fewer annotated objects than ten are filtered out. Finally, the new dataset ApolloScape+ consists of 3,011 training images, 703 validation images, and 1,200 test images. The new crowd and vehicle channel annotation operation is defined as Equations (10) and (11).

**Figure 6:**
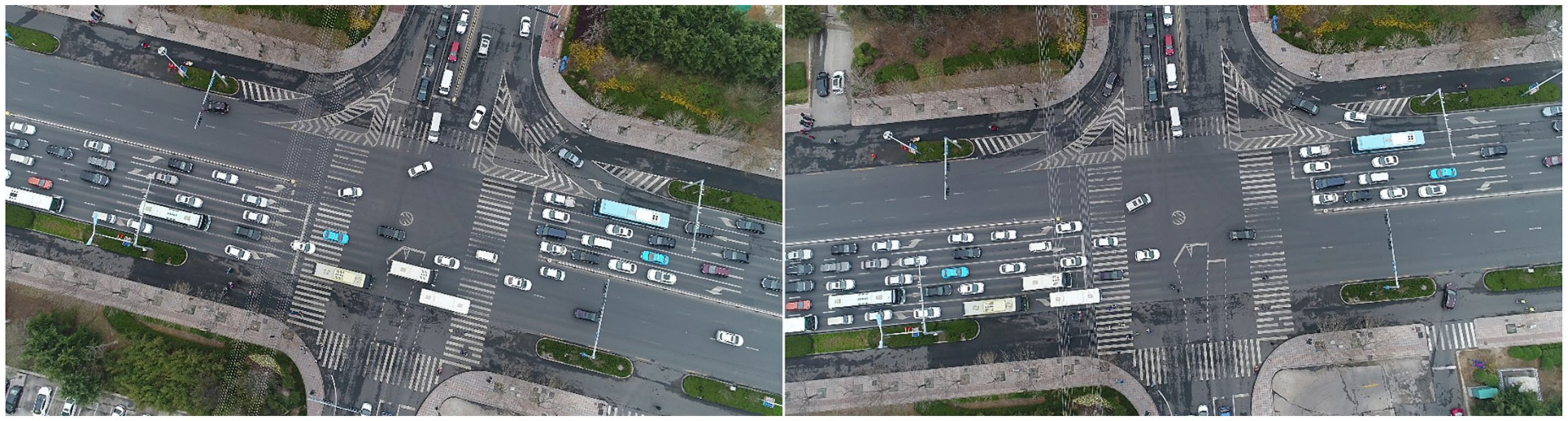
Images from the UAVDT dataset.

The UAVDT dataset is a vehicle dataset that can be used for target tracking and tasks such as object detection. The UAVDT is a dataset of videos and images taken by drones with a broader field of view, fewer changes in the scale and perspective of objects, but more dense objects, as shown in Figure 6. The dataset comprises 100 aerial videos, 40,000 images, and 841,500 annotations. In this paper, the category car and category bus in the original dataset are selected to form a 3D GT density map label. In the original dataset, the images with fewer annotated objects than ten are filtered out. Finally, the new dataset UAVDT+ consists of 6,023 training images, 1,208 validation images, and 2,103 test images. The new car and bus channel annotation operation is defined as Equation (11).

Evaluation metrics: Since the network’s final output is an estimated density map of two channels, this work uses the ordinary object counting standard evaluation metrics to test the predicted density map on each channel. The evaluation metrics include mean absolute error (MAE) and the root mean squared error (RMSE) (Siva et al., 2016). They are defined as follows: Equations (12) and (13):


(12) $$MAE=\frac{1}{N}\overset{N}{\underset{i=1}{\sum }}\left|C_{i}-C_{i}^{GT}\right|$$

(13) $$\mathrm{RMSE}=\sqrt{\frac{1}{N}\overset{N}{\underset{i=1}{\sum }}{\left(C_{i}-C_{i}^{GT}\right)}^{2}}$$

where *N* is the total number of test images, *C_i_^GT^* is the ground truth of counting, and *C_i_* represents the estimated count. Roughly speaking, MAE reflects the accuracy of the estimates, while RMSE reflects the robustness of the estimates.

### 5.2 Experimental setup

The multiple types of dense object counting network is built on Ubuntu 18.04 and Pytorch 1.11 experimental environments. The critical supporting hardware is Intel Core™ i7-12700 4.7 GHz and GeForceRTX3090. The model is trained using the *L_mul_* loss function and Adam optimizer, and the learning rate is 0.00001. The hyperparameter *λ_1_* and *λ_2_* of the joint multi-task loss *L_mul_* is set to 0.8 and 0.2. Data augmentation is used for training, where the original input image was randomly cropped to 1/2 of its original size and flipped horizontally. Accordingly, density maps used as training labels are cropped and flipped correspondingly. The affine transformation is used to make the size of labels consistent with the training output image size. The FLOPs of the model is 48.6 GFLOPs.

### 5.3 Comparisons with state-of-the-art

Table 2 shows the comparison results of our method with other methods on the VisDrone+ dataset. It is a challenging dataset with severe object occlusion and different perspectives. The existing methods, such as Li et al. (2018, 2020, 2023) and Fu et al. (2023), have performed well. We perform counting experiments on the existing methods by using images of people and vehicles respectively, while our method can simultaneously count people and vehicles in two channels. Our method significantly improves in MAE and RMSE on both crowd and vehicle channels, outperforming other state-of-the-art methods, and is more efficient without having to do two kinds of object count. It can also be found that the MSCNet and our method are close to each other in MAE and RMSE values. It is because of the use of improved functional modules referencing MSCNet in our method, and these functional modules can play an important role in object counting and classification in complex traffic environments. At the same time, our method has improved the loss function, and the counting precision is higher than that of MSCNet.

**Table 2:** Comparison with other methods on the visdrone+ dataset.

Methods	MAE		RMSE	
	People	Vehicle	People	Vehicle
CSRNet	12.8	11.1	37.2	16.8
Bilateral counting network	12.1	11.4	37.1	16.5
CCST	11.2	10.3	35.3	15.1
MSCNet	10.8	8.3	35.1	13.2
**Ours**	**10.3**	**7.8**	**34.7**	**12.2**

The bold value indicates the minimum error in the column.

Table 3 shows the comparison results of our method with other methods on the ApolloScape+ dataset. The traffic scenario of this dataset is highly complex, and the scale of the counting object varies widely. The experimental comparison shows that our method is superior to the other state-of-the-art methods (Li et al., 2018, 2020, 2023; Fu et al., 2023).

**Table 3:** Comparison with other methods on the ApolloScape+ dataset.

Methods	MAE		RMSE	
	People	Vehicle	People	Vehicle
CSRNet	13.3	11.8	39.3	18.9
Bilateral counting network	12.6	11.5	38.1	17.4
CCST	11.5	10.0	36.3	16.1
MSCNet	11.1	8.7	35.7	13.7
**Ours**	**10.9**	**8.2**	**35.9**	**12.7**

The bold value indicates the minimum error in the column.

Table 4 shows the comparison results of our method with other methods on the UAVDT+ dataset. The dataset is a UAV view dataset with a large field of view and a uniform object scale but with a high object density. To test the robustness of the multiple types of dense object counting model, we choose cars and busses as the targets of two-channel counting in this dataset. The experimental results show that our method can classify and count cars and busses at the same time, and it is better than the most advanced counting methods (Li et al., 2018, 2020, 2023; Fu et al., 2023).

**Table 4:** Comparison with other methods on the UAVDT+ dataset.

Methods	MAE		RMSE	
	Car	Bus	Car	Bus
CSRNet	14.1	13.7	19.1	18.8
Bilateral counting network	13.6	12.2	18.3	18.1
CCST	13.4	12.9	17.8	17.3
MSCNet	12.9	12.7	16.7	16.1
**Ours**	**12.1**	**11.2**	**15.9**	**15.3**

The bold value indicates the minimum error in the column.

This paper also conducts a comparative experiment on the performance of single channel counting on the ShanghaiTech dataset (Zhang et al., 2016), as shown in Table 5. This dataset is a dense crowds dataset. It consists of the ShanghaiTech Part_A dataset and the ShanghaiTech Part_B dataset. The Part_A dataset, which includes 482 images with 241,677 people labeled, has highly crowded scenes. The Part_B dataset is a relatively sparse dataset of people, consisting of 716 images with a total of 88,488 people labeled. The existing methods (Gao et al., 2021, 2023; Wang et al., 2023; Yi et al., 2024) have achieved good counting performance. Our method is also robust in single-channel crowd counting. The counting performance of MAE and RMSE on the Part_B dataset is superior to the existing advanced methods, and the counting performance on the Part_A dataset is also excellent.

**Table 5:** Comparison with other methods on the ShanghaiTech dataset.

Methods	ShanghaiTech Part_A		ShanghaiTech Part_B	
	MAE	RMSE	MAE	RMSE
Gao et al.	144.6	200.6	16.0	24.7
DACC	112.4	176.9	13.1	19.4
LEDCrowdNet	74.6	118.6	8.9	14.1
DMCNet	58.46	**84.55**	8.64	13.67
**Ours**	**58.1**	91.5	**7.2**	**11.0**

The bold value indicates the minimum error in the column.

**Figure 7:**
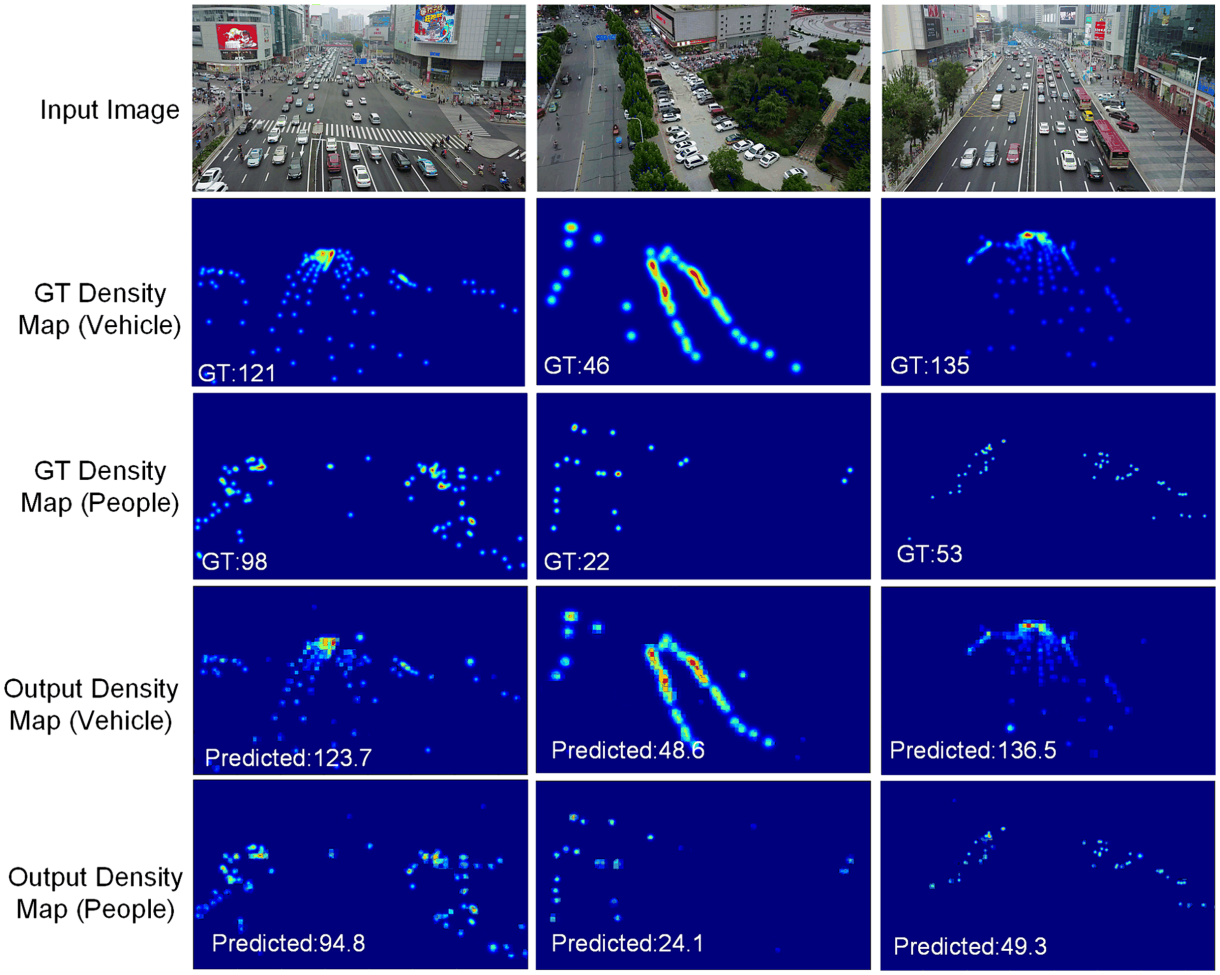
An output example of our method on the VisDrone+ dataset.

In addition to conducting comparative experiments on these datasets, we demonstrate the proposed method’s output on the VisDrone+ dataset, as shown in Figure 7. Three test images are selected, each containing dense vehicles and people in the traffic scenes. In Figure 7, the first row represents the input images of the counting network, the second row shows the ground truth density maps (GT maps) of the vehicle channel, the third row represents the GT density map of the people channel, and the fourth and fifth rows of images are the output (predicted density maps) of the vehicles and people. In addition, the bottom of the GT map and the predicted density map show the number of ground truth objects and the number of predicted objects.

### 5.4 Ablation study

In addition to comparing with state-of-the-art methods, in this section, we perform ablation studies on the VisDrone+ dataset and visualize the results, showing the effectiveness of our method, as shown in Figure 4. The different initial feature layers and whether to use the auxiliary task loss function are considered to configure the experiment. In this study, VGG and ResNet (Szegedy et al., 2017) are used as front-end layers because they have a good ability to extract features. The auxiliary loss function is not used to carry out counting experiments on people and vehicles, respectively. The detailed configuration information of the ablation study is as follows in Table 6.

**Table 6:** IFL and loss function ablation study configurations.

Configurations	Description
VGG-network (Ours)	The initial feature layer is VGG, our counting method, representing the channel of the crowd
	The initial feature layer is VGG, our counting method, representing the channel of the vehicle
ResNet-network	The initial feature layer is ResNet, representing the channel of the crowd
	The initial feature layer is ResNet, representing the channel of the vehicle
VGG-network-no-auxiliary	The initial feature layer is VGG, which does not use the auxiliary loss function, the method of counting only the crowd
	The initial feature layer is VGG, which does not use the auxiliary loss function, the method of counting only the vehicles

**Figure 8:**
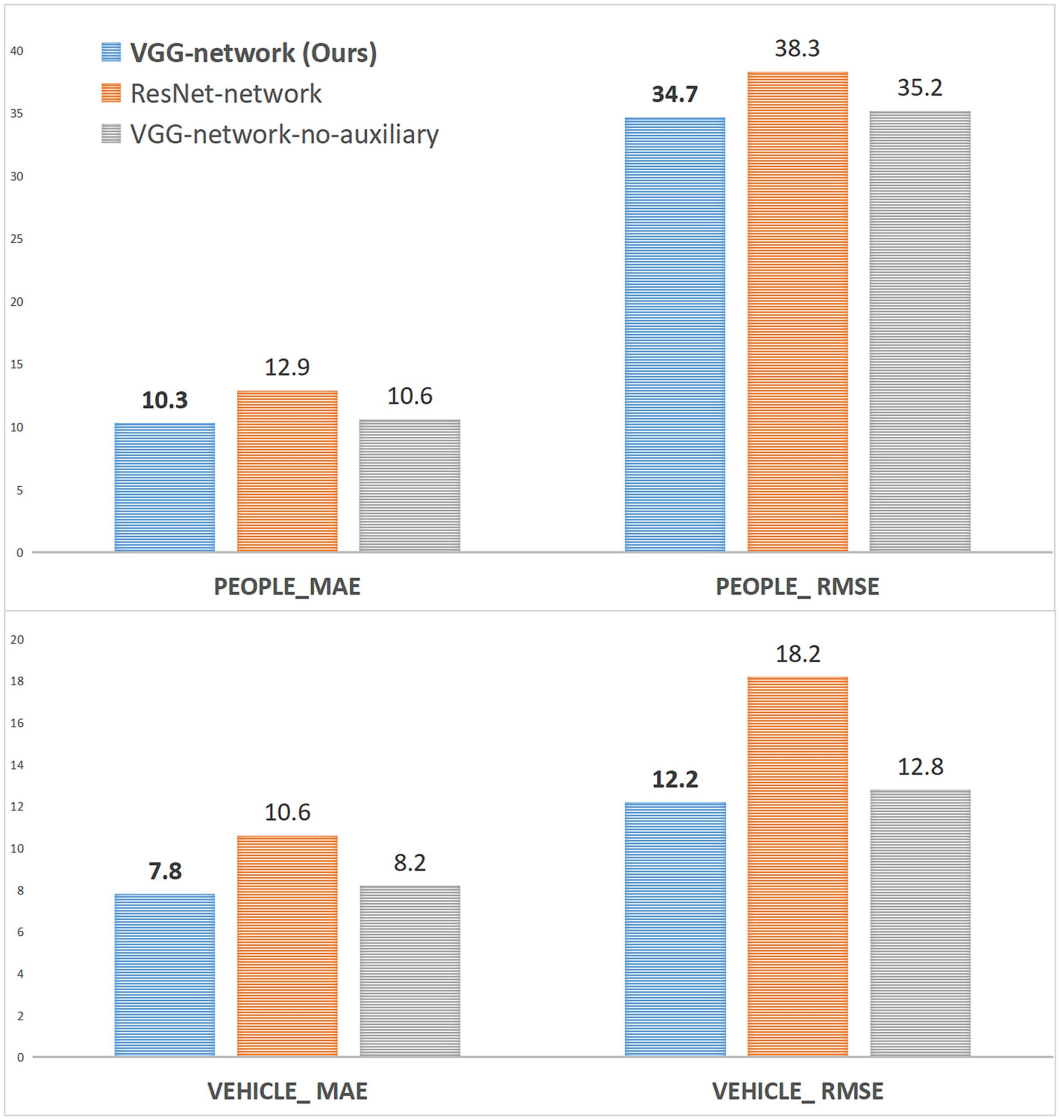
The ablation study of IFL and loss function on the VisDrone+ dataset.

From Figure 8, replacing VGG with ResNet for both crowd and vehicle channels does not achieve better count accuracy and even leads to lower MEA and RMSE. VGG has a strong transfer learning ability and is used by most of the dense object counting models in the backbone or front-end network architecture. We use it in the IFL to improve the efficiency of model training. Through ablation studies, it is found that VGG can achieve better counting performance than ResNet as the IFL in our method. In the face of object-dense and complex traffic scenes, the depth structure of ResNet loses more valuable and essential counting feature information. The analysis may be because the initial feature map extracted after downsampling 32x on ResNet is half more minor than after downsampling 16x on VGG. However, a feature map size that is too small will significantly impact the counting performance. The configuration “VGG-network-no-auxiliary” experiment confirms the effectiveness of using the auxiliary task loss function, which can achieve classification supervision of the model to complete the two-channel density map regression task and improve the counting performance under the single channel. Counting networks trained with the auxiliary loss function have higher counting accuracy (MAE and RMSE) on both the people and vehicle channels than when the people or vehicles are counted alone without the auxiliary loss function.

We also conducted an ablation study experiment on whether to use the SCM and CSM modules in FEL, and the experimental configuration is shown in Table 7.

**Table 7:** SCM and CSM ablation study configurations.

Configurations	Description
Counting network (Ours)	Our counting network, representing the channel of the crowd
	Our counting network, representing the channel of the vehicle
Network-no-SCM	Without SCM, representing the channel of the crowd
	Without SCM, representing the channel of the vehicle
Network-no-CSM	Without CSM, representing the channel of the crowd
	Without CSM, representing the channel of the vehicle

**Figure 9:**
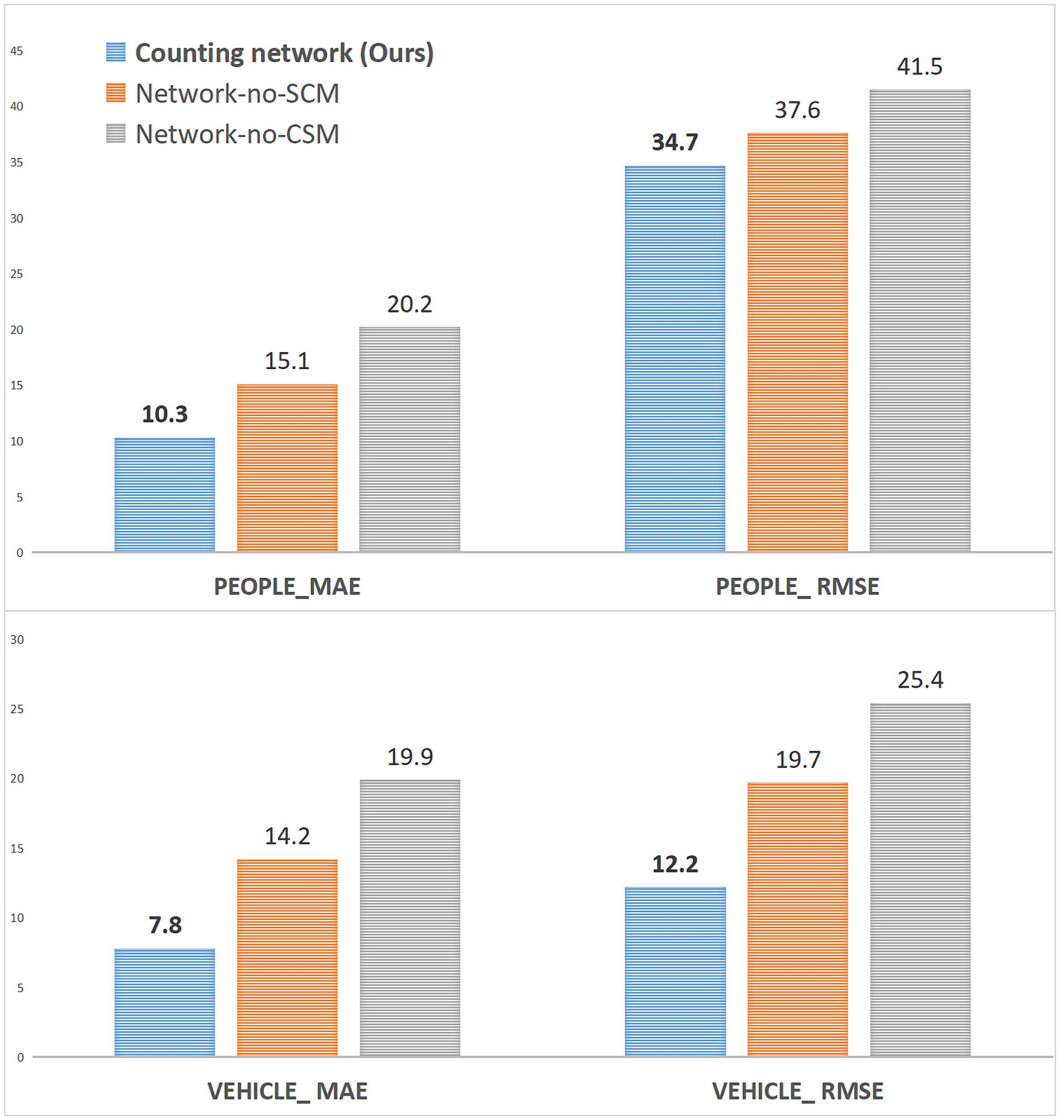
The ablation study of FEL on the VisDrone+ dataset.

As shown in Figure 9, when the SCM module is not used, the counting performance of the vehicle and crowd channels is decreased, while the counting performance of the two channels is greatly decreased when the CSM module is not used. The analysis is mainly due to the enhancement of the spatial correlation information by the SCM module, and this spatial feature is the primary feature information that needs to be extracted for the density map regression of the Regsubnet. The loss of the enhanced information on this part of the features will lead to an inevitable decline in counting performance. The CSM module mainly enhances the channel saliency feature to activate the important classification information in the channel. The loss of this part of the feature information will lead to a significant decline in the quality of the prediction density map classified by the channel.

## 6 Conclusion

This paper proposes a novel multiple types of dense object counting method for people and vehicle counting, which enhances the features of dense vehicles and people in complex traffic environments and supervises the classification of counting objects by channel. The method completes the density map regression counting of people and vehicles and realizes the simultaneous counting of two types of dense objects in complex traffic scenes. Among them, the FEL is used to enhance the features of vehicles and people, two types of dense objects in complex traffic environments, and extract more profound spatial correlation and channel salience information. To solve the problem of crowd and vehicle classification, we propose a joint loss function *L_mul_*, which supervises the classification and counting of the objects by channel. In addition, the function fully considers the spatial distribution information between pixels. It makes up for the spatial correlation lost by the main task of density map regression based on the Euclide-distance loss function *L_cou_*. The experimental results based on three datasets show that the innovative method classifies dense objects by channel and has better counting performance than the state-of-the-art methods. In the future, this work will continue to improve the feature extraction capability of mixed scenes and complete the simultaneous counting research of more than three types of dense objects.

Combined with the counting requirements of a complex traffic environment, we have studied existing problems, such as perspective change effects, scale variation, complex background environment, and complex foreground objects, and the corresponding solutions are proposed. The main strengths of this proposed method are summarized as follows:

1. The spatial correlation and channel salience features of dense objects in complex traffic scenes are enhanced using the FEL of SCM and CSM. In the spatial dimension, the counting object’s continuous large-scale scale variation and perspective change are solved. In the channel dimension, the salience coefficient of each channel is obtained by learning. This salience coefficient is mainly used to enhance the classification features of the foreground objects to achieve the counting of two types of objects and to enhance the extraction ability of counting features in complex backgrounds.
2. A new joint loss function *L_mul_* is designed to achieve the classification supervision task of the two objects. It not only completes the classification of targets according to channel but also improves the counting performance of the model in a single channel. This function takes complete account of the spatial correlation information between pixels. It makes up for the loss of spatial correlation information caused by using the Euclidean distance loss function *L_cou_* alone in the counting main task.

Although this research has achieved some success, there are still weaknesses. The multi-dense object counting network proposed in this paper can complete the feature extraction of two types of objects: vehicles and people. However, the “people” category includes not only pedestrians but also bicyclists and drivers of electric mopeds. These objects usually have weaker spatial correlation and little feature difference. The research needs to improve further the feature extraction ability of the model in complex traffic scenes, classify and count a variety of dense targets such as cars, pedestrians, and non-motor vehicles, which can be combined with fine-grained image classification, and optimize the formation process of training labels.

## Data availability statement

The original contributions presented in the study are included in the article/supplementary material, further inquiries can be directed to the corresponding author.

## Author contributions

QF: Conceptualization, Data curation, Formal analysis, Investigation, Methodology, Project administration, Software, Validation, Visualization, Writing – original draft, Writing – review & editing. WM: Funding acquisition, Methodology, Resources, Supervision, Writing – review & editing. WS: Data curation, Validation, Writing – review & editing. CP: Project administration, Validation, Visualization, Writing – review & editing.
